# Secretary’s Report Georgia State Commission on Tuberculosis

**Published:** 1905-07

**Authors:** Bernard Wolff

**Affiliations:** Secretary


SECRETARY'S REPORT GEORGIA STATE COMMISSION ON TUBERCULOSIS.

APRIL 18, 1905.

Your Secretary begs leave to present the following preliminary report: The Georgia State Commission on Tuberculosis was created for the specific purpose of ascertaining the status of tuberculosis in the State and of recommending the adoption on the part of the State of such means and measures as would tend to limit the spread and to suppress the disease. As a result of the organization meeting held in Macon, October 19, 1904, the Commission came to realize that the task set before it was a large one, and that the difficulties and obstacles in the way of its successful fulfillment were many and varied. The Commission was hampered at the outset by entire lack of pecuniary resource other than that voluntarily subscribed by the individual members in pursuance of a resolution introduced at the Macon meeting. This assessment, nearly all of which was promptly paid, amounted to $95.00, and was to be devoted to the necessary expenses incurred by the Secretary in the general work of the Commission. It was with this small and totally inadequate sum that the task of gathering statistics throughout the eleven Congressional districts in the State was undertaken.

The drafting of some specific form for distribution to the physicians in the State to secure from them the desired statistical data was next considered, and after consultation with the Chairman, the form appended was adopted. There has been some criticism offered concerning the comprehensiveness and usefulness of the form, but as the duty of the Commission was prescribed by the Act creating it, it was deemed expedient to adhere to the letter of the resolution, and in consequence the inquiry is designed only to cover the five propositions enumerated in the Bill, all other interrogatories having been deleted therefrom. To make this point clear, the Act is herewith inserted.

*Resolved by the Senate, the House concurring, That the Governor of the State of Georgia be, and he is hereby, authorized to raise a Medical Commission to consist of one physician from each Congressional district and ten from the State at large, who shall make a thorough investigation touching the propositions hereinafter set forth and numbered, and embody the same in a report to be submitted to the next session of the General Assembly:* (1) *The number of cases of tuberculosis in the State of Georgia.* (2) *The annual number of deaths from tuberculosis in the State of Georgia.* (3) *The number of cases contracted in the State and the number imported.* (4) *The measures that have been taken in the various localities for preventing the spread of the disease.* (5) *Whether or not the physicians of the State are in favor of taking measures for its prevention.*

*Be it further enacted, That the Commission shall serve without compensation and without entailing any expense whatever upon the State of Georgia in connection with the said investigation.*

The work of gathering statistics was proceeded with as follows: The number of physicians, without reference to school affiliation, in the eleven Congressional districts, was roughly estimated, and a sufficient number of blanks was furnished each district member of the Commission to cover his allotted territory. The various agencies by means of which the inquiry could be furthered were utilized, and each member of the Commission from the Congressional districts was requested to make a roster, compiling it from any and all authentic sources of information of the physicians residing in his district. This task was aided by official maps which were presented by the Assistant Commissioner of Agriculture, the Hon. R. A. Wright, to whom the appreciative thanks of the Commission are hereby tendered. It was suggested that the blanks be distributed by mailing them with a stamped and addressed envelope enclosed for reply. As the expense of this method of distribution had to be borne by the sender individually, it was not made mandatory, but was offered as the best means of securing prompt replies. The plan was adopted by the majority of district commissioners, and it is entirely likely that the modicum of success attained in collecting the data was due to it. The members of the Commission from the State at large were requested to co-operate with the district members in their work when called upon to do so. This they cheerfully consented to do, and in some instances have rendered most valuable aid.

In January, 1905, at the suggestion of the Chairman, the Secretary wrote a circular letter to the district members asking for a preliminary report in order to form some conception of the progress of the work, and to indicate its further course. The response was not as general as might have been desired, but was sufficient to show from the tentative reports that the returns were utterly disproportionate to the number of inquiries sent out. This was not attributed to indifference on the part of the profession generally toward the objects of the Commission, but rather to some other cause, procrastination or difficulty in sifting from inaccurate or unmethodically-kept records or supplying from memory cases about which information was desired.

At this time it became apparent that the scope of the Commission should be appropriately limited to the task of collecting statistics foregoing any enlargement of function. The slow and tedious process of gathering information by a canvass of physicians gave every promise of consuming most of the time allotted to the Commission, and the discouraging meagerness of response from these, the only dependable and available sources of information, led to the conviction that the function of the Commission for the present should rely solely in the acquisition of facts touching the numerical position of tuberculosis in the State. The promulgation to the public of instructive literature on the subject of the disease through the usual channels of diffusion was abandoned, at least for the time, or relegated to another body of somewhat similar intent. The preliminary reports were retained by the Secretary, and received such additions and emendations as were sent in until the time approached for this meeting, when they were all collected and epitomized, as will appear from the table. The Secretary desires to express sincere thanks to Dr. Alex. Mack for his valued assistance in tabulating the reports.

The foregoing statistics, while far from complete or accurate, are sufficient to indicate the wide prevalence of consumption in the State of Georgia, and the towering menace offered to the usefulness and health and life of its citizens. It quietly and steadily claims more victims annually than die during times of epidemic or pestilence, and is itself a pestilence in a wide sense, but one, unfortunately, with which familiarity has bred contempt of its danger. Nothing is done to stay its course or repress its spread. Every victim is a center from which irradiates a malevolent influence, of extent beyond conjecture, of the millions of germs discharged from his infected lungs in a single day, each one is fraught with the greatest possibility of evil, for each one is endowed with every element of self-propagation, and but needs favorable soil for growth and development to implant the disease in the body of a sound person.

The economic loss to the State through deaths from consumption can only be approximated. According to the statistics collected by this Commission, the deaths from tuberculosis in the State of Georgia, between the ages of twenty and forty, were about 981. Apply to this the statement of Hermann M. Biggs, M.D., General Medical Officer of the New York City Board of Health: "It may be a conservative estimate that each human life, at the average age at which tubercular deaths occur, is worth to the State $1,500. The cost of each life at this age is usually more than this." He says further: "We may assume that for a period of at least nine months these persons are unable to work, and must be cared for. The loss of this service during this period may be estimated at one dollar a day." Add this to the amount by death, at rate of one dollar per day for two hundred and seventy days, yields a total of $1,731,370. These figures have to do with those who die, and do not take into consideration those who recover from the disease; though the number of the latter is not obtainable, it is easily one-third of those who die and to this sum must be added that loss by withdrawal from work during illness of those who recover. Furthermore, the economic waste incident to the care and nursing of consumptives, the drain upon charitable resources of tuberculous indigent families, the care by church or other institutions of dependent orphan children—all these elements of the problem must be considered in summing up the huge total of moral and monetary loss to the State occasioned by the disease.

There is still another aspect of the question, and that is its relation to life insurance. Three of the largest companies give the percentages of deaths among policy-holders as follows: 9 per cent.; 22 per cent.; $8\frac{1}{2}$ per cent. A reduction of this mortality would directly affect the cost of life-insurance premiums, and policy-holders be proportionately benefited.

There are three propositions connected with the study of tuberculosis which are incontestably established, and of which the practical application is one of the chief objects of the recommendations of this Commission:

1. Consumption is a communicable disease, directly acquired by a healthy person from a human being or an animal suffering with the disease. The contagious principle is a germ—the tubercle bacillus—which escapes from the body of a person or animal suffering from the disease, with the secretions, and is immediately conveyed to the body of another person by food or air, or it is deposited upon the ground, and becoming mixed with dust, is taken into the system and produces the disease. This disease is invariably produced in this manner, and in this manner alone. Without the tubercle bacillus there is and can be no consumption. Consumption is always true to type, just as with similar phenomena in the vegetable kingdom. An oak will always grown from an acorn—a pine never.

2. Tuberculosis is a preventable disease. This follows as a corollary from the preceding proposition. If the provoking cause of a disease is known and established, a very long step is at once made in the direction of preventing the disease. The actual cause of tuberculosis is the tubercle bacillus. It is thoroughly well-known by what avenues the germ enters the body, how its growth and development are influenced and modified by circumstances, both within and without the body, and how it is actively impaired, lessened or suppressed by chemical, dietetic and hygienic agents and means. To accomplish these last named results, a double purpose is subserved—the relief of the individual from the disease which has fastened upon him, and the protection of others exposed to it. The protection of others constitutes the great end and object of organized systematic movements against the disease. The germs destroyed or de-energized, as they leave the body of the infected individual, so that they are rendered harmless and unfit for their baneful mission, and surrounding the victim who is the host of the germs in a state of activity, by all those influences which tend to increase his inherent resistance against the parasites' mastery over him, and his ability to overcome it, by heightening the efficiency of other organs to compensate for the curtailment in function of those concerned in the diseased process. These are cardinal principles and chief purposes of the anti-tuberculosis crusade.

It is very questionable whether consumption is ever directly inherited; that is, that the child is born with the disease already existing in its body. There is, however, a predisposition which is frequently a matter of hereditary transmission, but it is in effect no more than a weak or debilitated state of the body which lowers its resistance to the disease. Heredity may therefore be practically eliminated as a factor of moment in the production of consumption. To attempt to prevent the disease it is necessary to limit the amount of infection in the first instance. Here arises a situation calling forth the exercise of delicacy and tact. It has been said with propriety that while we must insist upon the enforcement of sanitary laws, yet we must avoid creating in the minds of people an unnecessary fear which would work undue hardship upon those afflicted. It is not the object nor design of the restrictive measures to surround the unfortunate victim of consumption with such a hedge of hampering exactions as will make existence irksome to him, or to have him regarded as anciently were the lepers. A few simple sanitary rules should be suggested, and if necessary enforced, to protect others against infection, and these need not compromise personal liberty nor arouse fear and aversion. Prevention may be almost summarized in one phrase—the disinfection of tuberculous sputum.

3. Tuberculosis is a curable disease. The time has passed when consumption was viewed as a disease which once established had but one termination—death. The average number of recoveries, as gathered from statistics under modern methods of treatment, is forty per cent. If taken early and actively, sixty-five per cent. recover. The curability of consumption must not, however, be considered too lightly. It represents the collection and utilization of all natural forces and artificial means of defense to be brought into play in the contest against the disease. Prevention and cure are to be obtained only in the manner indicated in the recommendations of this Commission.

In view of the foregoing facts the Commission respectfully makes the following recommendations:

1st. Compulsory notification of tuberculous disease.

2d. Rigid enforcement of already existing local ordinances against spitting, emptying cuspidors, etc.

3d. Disinfection of rooms or houses occupied by consumptives at stated intervals.

4th. Isolation or segregation of consumptive cases in public institutions—the asylum, workhouses, prisons.

5th. Persistent education of the public in all matters pertaining to the nature and prevention of tuberculosis.

6th. That the Governor be authorized to appoint a commission consisting of five persons to investigate the feasibility and desirability of establishing a sanatorium for the care ot consumptives in the State of Georgia. That the sum of One Thousand Dollars ($1,000) be appropriated out of the general revenue of the State to defray the expenses of said Commission.

That the said Commission report to the General Assembly at its next meeting.

Respectfully submitted,

BERNARD WOLFF,
Secretary.

---

GOVERNOR PENNYPACKER, of Pennsylvania, has signed the bill passed by the Legislature making it a misdemeanor to use more than one per cent. of boric acid compounds for the preservation of food commodities.

